# Overview of best practices for buprenorphine initiation in the emergency department

**DOI:** 10.1186/s12245-024-00593-6

**Published:** 2024-02-19

**Authors:** Terence Hughes, Nicholas Nasser, Avir Mitra

**Affiliations:** 1 The Mount Sinai Hospital, 1 Gustav Levy Place, New York, NY 10029-6574 USA; 2 Mount Sinai Beth Israel, 281 1st Ave, New York, NY 10003 USA

**Keywords:** Social emergency medicine, Addiction medicine, Harm reduction, Buprenorphine, Opioid use disorder

## Abstract

In recent decades, opioid overdoses have increased dramatically in the United States and peer countries. Given this, emergency medicine physicians have become adept in reversing and managing complications of acute overdose. However, many remain unfamiliar with initiating medication for opioid use disorder such as buprenorphine, a high-affinity partial opioid agonist. Emergency department-based buprenorphine initiation is supported by a significant body of literature demonstrating a marked reduction in mortality in addition to increased engagement in care. Buprenorphine initiation is also safe, given both the pharmacologic properties of buprenorphine that reduce the risk of diversion or recreational use, and previously published literature demonstrating low rates of respiratory depression, sedation, and precipitated withdrawal. Further, barriers to emergency department-based initiation have been reduced in recent years, with publicly available dosing and up-titration schedules, numerous publications overviewing best practices for managing precipitated withdrawal, and removal of USA policies previously restricting patient access and provider prescribing, with the removal of the X-waiver via the Medication Access and Training Expansion Act. Despite reductions in barriers, buprenorphine initiation in the emergency room remains underutilized. Poor uptake has been attributed to numerous individual and systemic barriers, including inadequate education, provider stigma, and insufficient access to outpatient follow-up care. The following practice innovation aims to summarize previously published evidence-based best practices and provide an accessible, user-friendly initiation guide to increase emergency physician comfortability with buprenorphine initiation going forward.

## Background

Opioid overdoses pose one of today’s most significant public health crises, with fatalities in the USA increasing by tens of thousands per year since 2019, and now exceeding 80,000 as of 2021 [1]. Curbing this increase, and ultimately decreasing opioid overdose fatalities, requires an urgent, multimodal response with coordination across sectors and stakeholders. Medication for opioid use disorder (MOUD)—such as methadone, buprenorphine, and naltrexone—is now widely considered the standard of care for patients interested in decreasing or halting recreational opioid use altogether. Compared to the previous standard of care, an abstinence-only approach, MOUD is both safe and effective at increasing long-term connection to care, blocking withdrawal symptoms and cravings, decreasing long-term harmful opioid use, and reducing overdose risk [2]. Moreover, in patients who presented to the emergency department after a non-fatal overdose, MOUD initiation has been demonstrated to significantly reduce mortality [3].

Buprenorphine is a partial mu-opioid receptor agonist and a weak kappa-opioid receptor antagonist [4]. Compared with other options for MOUD such as methadone, this mechanism of action is unique. As a partial agonist, buprenorphine has what is commonly called a ‘ceiling effect,’ with a decreased risk of unintended adverse events like respiratory depression, and potentially less euphoria with rising doses when compared to full agonists [5]. Further, buprenorphine has a high affinity for the opioid receptor, and can thereby displace or prevent the binding of other opioids; this property is thought to reduce the risk of return to use by blocking euphoria when co-ingested with lower affinity, recreational opioids [6]. Buprenorphine is available in a wide array of formulations, including oral tablets, subcutaneous extended release, subdermal implant, transdermal patch, and most commonly, sublingual film [7]. Sublingual buprenorphine is often co-formulated with naloxone with the aim of decreasing diversion and recreational use. Mechanistically, naloxone has poor sublingual but sufficient intravenous bioavailability. Therefore, its co-formulation theoretically decreases the risk of diversion for recreational use; however, data on its effect in this capacity is limited [8].

Buprenorphine as a maintenance medication for OUD has overwhelming evidence demonstrating its safety and efficacy [9]. Across care settings, buprenorphine prescribing for patients with OUD has consistently increased in the last decade; however, utilization remains low, with just 11.2% of patients with OUD receiving any type of MOUD in 2021 [10, 11]. This trend is driven by many barriers to buprenorphine prescribing and access including insufficient physician education, provider stigma against patients who use drugs and resulting health system mistrust, and poor access due to income and employment-based health coverage [12–14]. Buprenorphine uptake has been disparate across patient populations with particularly low utilization for patients of color and publicly insured or uninsured patients [15, 16].

Historically in the USA, buprenorphine access was limited by federal regulation, enshrined by the 2000 Drug Addiction Treatment Act (DATA 2000), which required physicians to obtain additional training in order to prescribe buprenorphine. In 2023, this act was rescinded and replaced with the broader Medication Access and Training Expansion Act (MATE), which requires all physicians who prescribe any controlled substance to complete a one-time eight-hour training [17, 18]. Despite the removal of DATA 2000 and the broadness of MATE, their combined legacy continues to restrict access with physicians feeling uncertain about prescribing or remaining unaware that they are now permitted to prescribe [19–21].

Further, buprenorphine initiation and additional care for patients with OUD have previously been regarded as the responsibility of outpatient psychiatrists, primary care doctors, and addiction specialists [22, 23]. However, access to these care settings is often inadequate. In contrast, the emergency room (ER) is always open and serves as a frequent health system touchpoint for many patients with OUD who lack access elsewhere. As a result, emergency medicine as a field is uniquely positioned to not only respond to acute overdoses, but also to initiate comprehensive care for this patient population, including buprenorphine [24].

Buprenorphine initiation has been noted in the emergency medicine literature as early as 2004, with a significant increase in focus in recent years [25, 26]. ER-based initiation is evidence-based as the seminal randomized clinical trial found increased engagement with outpatient addiction treatment and reduction in illicit opioid use among patients initiated on buprenorphine in the emergency setting compared to those provided with just a referral or brief intervention [27]. However, despite clear evidence of the benefits of this intervention in the ER, adoption of this practice remains relatively uncommon. The following paper aims to address this practice gap by overviewing ER-based buprenorphine initiation protocols, providing readers with a pragmatic guide to initiation, and exploring additional considerations that emergency providers should take into account.

## Buprenorphine initiation

Candidates for buprenorphine initiation in the ER include any patient with OUD who self-identifies that MOUD is in line with their individual care goals. This typically consists of patients who are striving to cut back on, or completely cease their recreational opioid use. Given inconsistent patient knowledge of and attitudes toward buprenorphine, all patients with OUD who present to the ER merit a conversation about MOUD availability and potential benefits, as well as the opportunity to ask questions to learn more.

Historical practice dictates that to initiate buprenorphine in the ER, patients should be experiencing moderate to severe withdrawal, which can be assessed using the Clinical Opioid Withdrawal Scale (COWS) [28]. Initiating buprenorphine in patients who are not yet in moderate withdrawal risks causing precipitated withdrawal symptoms (PWS) [29]. Though COWS is a clinically validated scale for detecting withdrawal, providers should continue to utilize patient perspectives to guide their identification of withdrawal, as patients are experts in identifying their own symptoms of withdrawal. Urine drug screening is typically recommended by treatment guidelines for buprenorphine initiation to help identify unreported drug use. However, urine drug screening can be harmful to patients due to high false positive and negative rates [30, 31]. Thus, providers should not routinely obtain a urine drug screen prior to buprenorphine initiation, and when a drug screen is ordered, should exercise clinical judgment and avoid rigid clinical conclusions using these results alone.

**Fig. 1:**
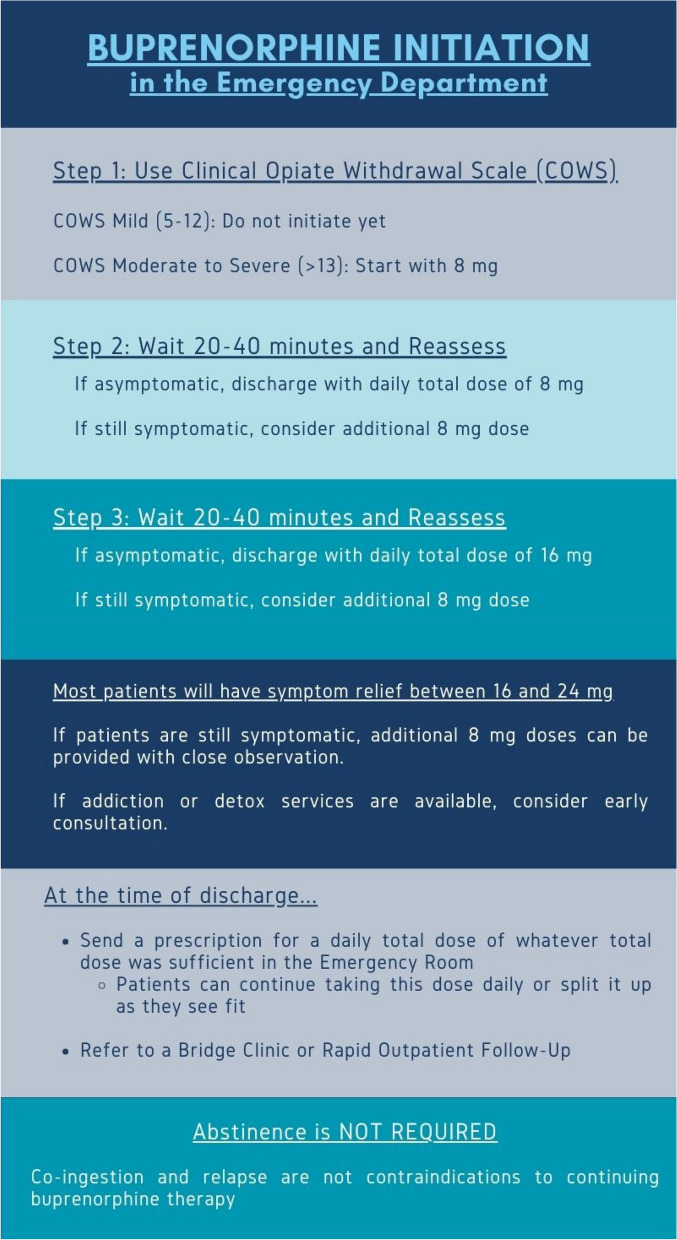
Buprenorphine initiation in the emergency department

For most patients, buprenorphine initiation starts at 8 mg administered sublingually. Patients should be encouraged to allow the sublingual film to dissolve completely before eating, drinking, or smoking and should not swallow the film. This is a starting dose and rarely will be sufficient alone, particularly in patients with higher tolerance due to regular consumption of higher opioid doses. For these patients, subsequent up-titration will be necessary to achieve stabilization. After this initial dose, patients should be observed for 20 to 40 min, with subsequent dosing in 8 mg intervals. In some cases, depending on the level of opioid dependence, patients may require total doses reaching 24–32 mg [32, 33]. Figure 1 illustrates a practical guideline for buprenorphine initiation, developed by the authors.

**Fig. 2:**
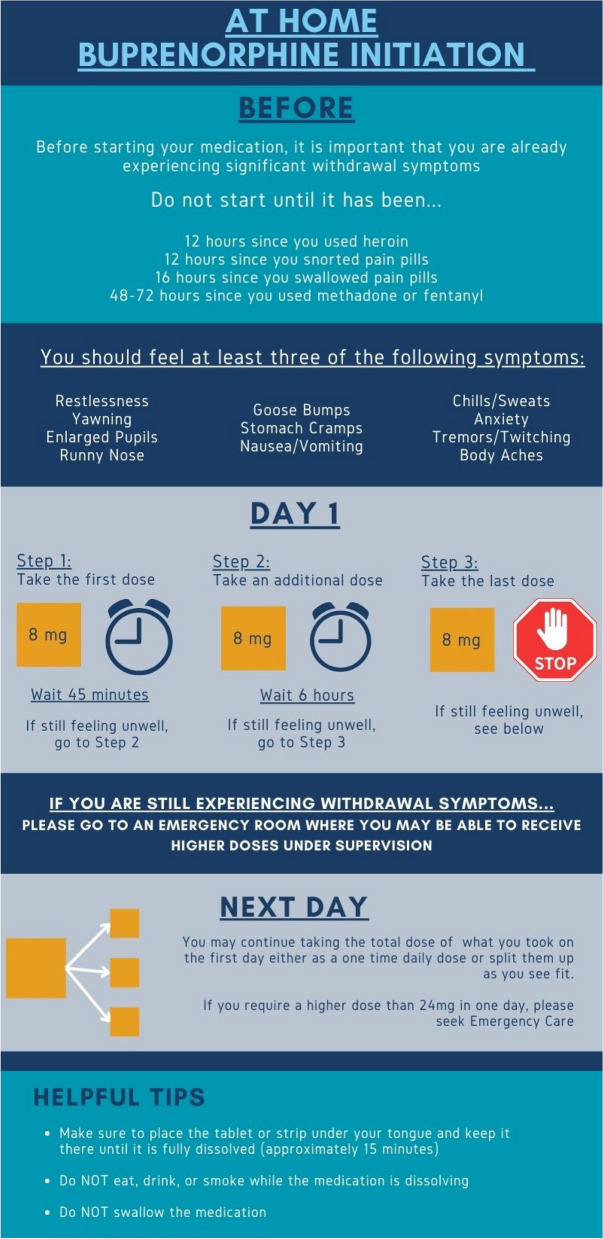
At-home buprenorphine initiation instructions

For patients not actively in withdrawal at the time of ER presentation, providers have two options: they may hold patients in the ER until withdrawal symptoms emerge and then initiate treatment on-site, or they can prescribe buprenorphine on discharge. In the latter case, patients are provided the autonomy to initiate buprenorphine outside of medical supervision in their space of choice. Randomized control trials have demonstrated that at-home buprenorphine induction protocols following ER discharge are both feasible and safe with low rates of PWS [34, 35]. Clear written and verbal instructions must be provided to maximize uptake and minimize adverse events. Patients should be instructed to refrain from self-initiating until moderate withdrawal symptoms commence, which will vary in timeline depending on individual metabolism, tolerance, and type of opioid(s) used. Once patients self-identify that they are in moderate withdrawal, they can start with an 8-mg sublingual film and self-titrate. An example of take-home patient instructions developed by the authors is illustrated in Fig. 2.

## Precipitated withdrawal management

PWS are difficult to distinguish from typical opioid withdrawal because the symptoms are the same. What defines PWS is the acute, abrupt worsening of symptoms within the hour following buprenorphine administration, contrasted with typical withdrawal which gradually worsens over the course of hours-to-days. The continued gradual worsening or persistence of withdrawal symptoms following a first dose of buprenorphine does not represent PWS, but rather an insufficient treatment of withdrawal symptoms requiring increased buprenorphine dosing. PWS are thought to occur mechanistically due to the partial mu agonist effects of buprenorphine. PWS are more likely in patients with a higher degree of opioid dependence, and decreased time between full agonist use and buprenorphine initiation [36]. PWS are rare in controlled studies; however, many patients commonly cite this experience as a concern when considering buprenorphine initiation [37]. Many patients may instead opt for alternative treatments or continue opioid use to avoid withdrawal symptoms. It is important for practitioners to proactively understand and address this fear by emphasizing the effective tools for treatment of PWS if they do occur.

**Fig. 3:**
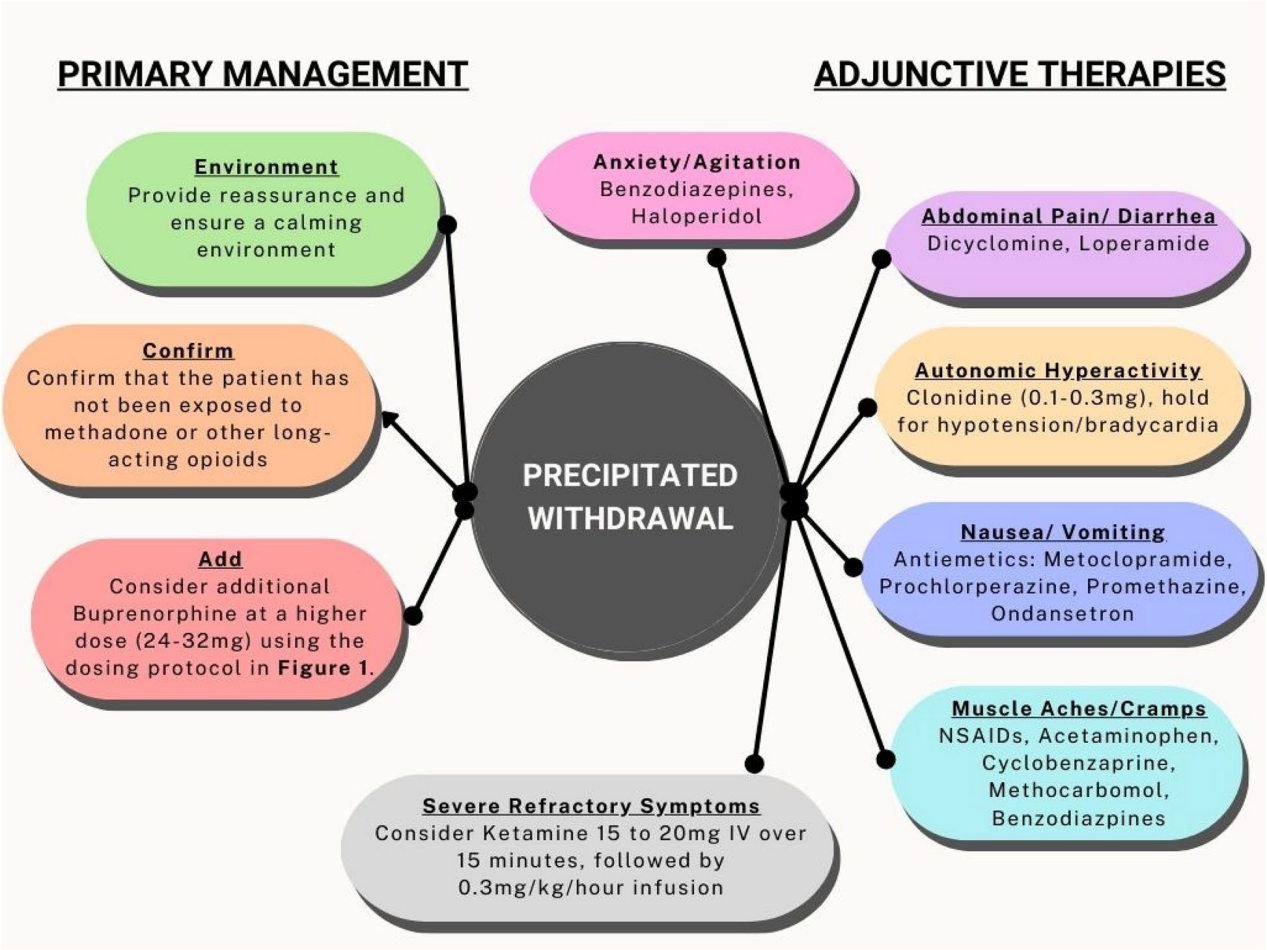
Precipitated withdrawal management strategies and therapies for emergency practitioners

When initiating buprenorphine, providers should first ask patients about prior experience taking buprenorphine. This provides providers with an opportunity to assess a patient’s prior PWS experience if applicable, provide patient education as needed, and leverage shared decision-making to decide on the best path forward. PWS can be managed primarily with additional increased doses of buprenorphine, often up to 32 to 64 mg, to saturate remaining unoccupied opioid receptors. Higher doses of buprenorphine have been demonstrated to be safe, without episodes of respiratory depression in a previously published case series [38]. In addition to increased buprenorphine doses, PWS can also be managed with adjunctive therapies targeted at withdrawal symptoms. For example, patients with nausea and vomiting can be effectively managed with antiemetics like ondansetron, metoclopramide, or prochlorperazine, while those with diarrhea can be managed with loperamide. Likewise, patients experiencing autonomic hyperactivity such as tachycardia, hypertension, diaphoresis, and hyperthermia can be managed with clonidine. Acute anxiety and agitation can be managed using benzodiazepines, antipsychotics, and ketamine if refractory to other treatment modalities. Lastly, patients with PWS often experience a heightened sense of pain, coined hyperalgesia, for which pain management adjuncts like NSAIDs are effective [39]. A schematic overview developed by the authors of these adjunctive treatment strategies to manage PWS is captured in Fig. 3.

It is important to note that patients already taking methadone and attempting to switch forms of MOUD to buprenorphine can have PWS up to 72 hours after their last use, given the long half-life of methadone. Because of this, buprenorphine initiation in this population requires more caution, with protocols specifically tailored to bridging between these two forms of MOUD [40, 41].

## Beyond the emergency room: prescribing buprenorphine with linkage to long-term care

Before considering initiation of buprenorphine, emergency clinicians should be keenly aware of what resources are available for their patients for follow-up care and should adjust their prescribing practice based on said resources. In the past, some practitioners preferred to administer each buprenorphine dose in the ER, requiring patients to follow up daily until a long-term prescriber was established. This practice is not recommended because it is time-consuming, resource-intensive, and increases patient-facing barriers to access and utilization.

Instead, we recommend discharging patients with a buprenorphine prescription once they are clinically stable and do not require additional medical work-up or management. Providers may simply prescribe a daily dose of 16 mg as some evidence supports that most patients feel improved at this dosage [32, 42]. We recommend being more flexible and tailoring discharge dosage to each individual; a common strategy to do this is to prescribe a daily dose equal to the total quantity required to stabilize the patient in the ED. Patients may opt to split their doses throughout the day or take the dose at once, depending on their individual preferences and experience of cravings, with both dosing schedules being safe and effective [40, 43].

Beyond the ER, rapid outpatient follow-up is essential to connect patients with outpatient buprenorphine prescribers and ensure the long-term sustainability of ER-based initiation. One model of doing this is providing follow-up with a known buprenorphine prescriber in the surrounding community, often a primary care physician or psychiatrist, and providing a bridge prescription until this appointment. However, this model of follow-up has been unreliable given the low availability of outpatient appointments and poor coordination between the ER and the community settings.

Bridge clinics are a novel, innovative model of post-ER care for patients with OUD that aims to decrease barriers to obtaining rapid outpatient follow-up and linkage to long-term OUD care. Evidence supports that bridge clinics have been successful in filling the gaps in the care continuum for this patient population; qualitative work suggests that this setting of care is acceptable to patients with OUD. Likewise, bridge clinics have been shown to reduce ER utilization and facilitate higher buprenorphine adherence [44–46]. For emergency clinicians without bridge clinic availability, we recommend close relationships with local outpatient providers who prescribe buprenorphine and the provision of a buprenorphine prescription that will bridge a patient to their intake appointment. In areas where outpatient buprenorphine prescriptions are currently unavailable, we recommend emergency clinicians find ways to advocate for the urgent creation of these critical care pathways.

## Additional considerations for emergency care providers

Beyond initiating buprenorphine and providing linkage to long-term care, emergency providers must be well versed in a number of additional considerations, including (1) harm reduction approaches and tools, (2) global and local practice variations, and (3) ongoing areas of investigation that could impact future best practices.

### Harm reduction approaches and tools

Harm reduction should be central to care for all patients who use drugs and patients with OUD in the ER [47, 48]. This starts when a provider asks a patient to identify how their opioid use impacts their lives and to share their goals of care—which may include continued, decreased, or complete cessation of use. From this starting point, the provider’s objective is to provide information and tools to minimize harm, while also facilitating that patient’s stated goal [49]. For those patients who identify complete cessation or decreased use as their goal, buprenorphine initiation in the ER is one tool to help achieve this.

All patients with OUD, regardless of interest in buprenorphine, should receive on-site provision of harm reduction supplies in the ER. This includes the provision of take-home naloxone, intended to reverse overdose in the community. Likewise, at-home drug testing strips that can detect fentanyl and xylazine can play a role for patients using substances with unclear contaminants or purity. Given nationwide trends in drug supply contamination, many patients will already anticipate, or know, that there is fentanyl or other analogues in their opioids. Accordingly, the utility of distributing test strips will vary on a case-by-case basis [50–54]. These supplies should be provided with clear patient education regarding how patients can use these tools to minimize harm.

In addition, this patient population should also receive harm reduction education and connection to relevant community resources from the ER. As the availability of resources outside of each emergency department can be quite varied, providers should be keenly aware of the facilities in their own community. Emergency providers can discuss best practices that reduce infection risk and provide information about local syringe exchanges for those patients intending to continue injection use [55]. Similarly, for patients who risk using alone, emergency providers can encourage the utilization of overdose prevention centers where available, and telephone or messaging-based never-use-alone platforms to minimize overdose risk [56–60].

### Global and local practice variations

Buprenorphine initiation best practices shared in this paper, and much of the evidence published in the prior literature, stems from urban, academic sites in the USA. As such, practitioners in rural, low-resource, or global settings will have their own unique considerations to take into account.

Importantly, ER-based buprenorphine initiation has unique advantages in rural areas when compared to methadone. In many rural areas, methadone can be a particularly challenging and inaccessible form of MOUD due to the significant distances rural patients must travel each day to reach their methadone clinic [61]. Despite this advantage of buprenorphine, emergency providers working in rural settings will still face unique challenges compared to urban providers when it comes to linkage to long-term care and connection with harm reduction-focused community-based organizations [62–64]. Some solutions to these uniquely rural challenges have already been proposed and trialed—including telehealth-based buprenorphine prescriptions and partnerships with advanced practice providers such as nurse practitioners, physician assistants, and pharmacists who may be more available in rural settings [65, 66]. While these implementations show promise, there remains much to be done to continue increasing buprenorphine availability from the ER and beyond in rural settings.

The majority of evidence for buprenorphine is USA-focused because the USA has the highest overdose mortality in the world. Despite this, buprenorphine initiation and other implementations focused on caring for patients who use drugs in the emergency setting could have important global implications. For example, one study modeled the potential impact of MOUD uptake and retention on mortality globally and found significantly greater survivorship benefits in low- and middle-income countries, likely due to a reduction in mortality associated with HIV and other communicable diseases [67].

### Ongoing and future areas of investigation

Beyond the best practices shared in this review, there is much ongoing research involving buprenorphine initiation in the ER that has the potential to change practice in the future. Here, we highlight a non-comprehensive sampling of ongoing investigation, to encourage emergency providers to iteratively update their practice as this evidence base continues to develop.

First, a poorly regulated drug supply has resulted in an increased presence of potent synthetic analogs like fentanyl and nitazenes [68–70]. A growing body of qualitative and mixed methods literature has reported growing concern from both patients and practitioners that this evolving drug supply necessitates changes to buprenorphine initiation protocols [71–74]. Some postulate that PWS following buprenorphine initiation for people who use fentanyl can happen even after periods of abstinence greater than 24 hours, given fentanyl’s lipophilicity [75, 76]. However, another retrospective cohort study found no significant difference in PWS between starting buprenorphine doses for patients using fentanyl compared to those who did not [77]. Taken together, these inconsistencies highlight the need for additional research to determine whether, and how to modify buprenorphine initiation protocols for patients regularly using more potent opioid analogs.

Many patients cite fear of PWS as a primary reason for not initiating buprenorphine; thus, dosing interventions targeted at minimizing this risk have the potential to be particularly impactful [78]. Accordingly, other areas of investigation have proposed various ways to alter buprenorphine initiation protocols, with the goal of increasing patient comfort and driving long-term adherence. For example, numerous case reports and narrative reviews have proposed buprenorphine micro-induction protocols, which bypass the initial period of waiting for the patient to enter withdrawal and instead immediately start at a low dose (0.2–0.5 mg) with subsequent increases over the course of hours-to-days [79–85]. Another example of dosing modification can be found in a published case report that details intentional precipitated withdrawal with self-administered naloxone, followed by rapid induction of high-dose buprenorphine [86]. This case study has high potential applicability to the ER, where patients often arrive in withdrawal after receiving naloxone in the field. Most studies of these alternative dosing protocols have been done in the inpatient, primary care, or community settings—so much remains to be learned about potential applications in the emergency care setting.

## Conclusion

Buprenorphine is a high-affinity partial opioid agonist that is clearly demonstrated to improve clinically relevant outcomes for patients with OUD. Buprenorphine initiation in the ER is both safe and effective, particularly when followed by linkage to long-term care. However, the uptake of this best practice has been inadequate to date. This practice innovation aims to increase ER-based buprenorphine initiation on the ground by providing a user-friendly guide that reviews best practices, other considerations, and potential future areas of practice change. In highlighting buprenorphine initiation as one evidence-based intervention, we hope to more broadly emphasize that emergency practitioners have a critical role to play in responding to the worsening opioid overdose public health crisis.

## Data Availability

Data sharing is not applicable to this article, as no datasets were generated or analyzed.

## Abbreviations

USA: United States of America
OUD: Opioid use disorder
MOUD: Medications for opioid use disorder
DATA 2000: Drug Addiction Treatment Act
MATE: Medication Access and Training Expansion Act
ER: Emergency room
COWS: Clinical Opioid Withdrawal Scale
PWS: Precipitated withdrawal symptoms
